# War-induced collapse and asymmetric recovery of large-mammal populations in Gorongosa National Park, Mozambique

**DOI:** 10.1371/journal.pone.0212864

**Published:** 2019-03-13

**Authors:** Marc E. Stalmans, Tara J. Massad, Mike J. S. Peel, Corina E. Tarnita, Robert M. Pringle

**Affiliations:** 1 Department of Scientific Services, Beira, Sofala Province, Mozambique; 2 ARC-Animal Production Institute, Rangeland Ecology Group, Nelspruit, South Africa; 3 Department of Ecology & Evolutionary Biology, Princeton University, Princeton, NJ, United States of America; U.S. Geological Survey, UNITED STATES

## Abstract

How do large-mammal communities reassemble after being pushed to the brink of extinction? Few data are available to answer this question, as it is rarely possible to document both the decline and recovery of wildlife populations. Here we present the first in-depth quantitative account of war-induced collapse and postwar recovery in a diverse assemblage of large herbivores. In Mozambique’s Gorongosa National Park, we assembled data from 15 aerial wildlife counts conducted before (1968–1972) and after (1994–2018) the Mozambican Civil War (1977–1992). Pre-war total biomass density exceeded 9,000 kg km^-2^, but populations declined by >90% during the war. Since 1994, total biomass has substantially recovered, but species composition has shifted dramatically. Formerly dominant large herbivores—including elephant (*Loxodonta africana*), hippo (*Hippopotamus amphibius*), buffalo (*Syncerus caffer*), zebra (*Equus quagga*), and wildebeest (*Connochaetes taurinus*)—are now outnumbered by waterbuck (*Kobus ellipsiprymnus*) and other small to mid-sized antelopes. Waterbuck abundance has increased by an order of magnitude, with >55,000 individuals accounting for >74% of large-herbivore biomass in 2018. By contrast, elephant, hippo, and buffalo, which totaled 89% of pre-war biomass, now comprise just 23%. These trends mostly reflect natural population growth following the resumption of protection under the Gorongosa Restoration Project; reintroductions (465 animals of 7 species) accounted for a comparatively small fraction of the total numerical increase. Waterbuck are growing logistically, apparently as-yet unchecked by interspecific competition or predation (apex-carnivore abundance has been low throughout the post-war interval), suggesting a community still in flux. Most other herbivore populations have increased post-war, albeit at differing rates. Armed conflict remains a poorly understood driver of ecological change; our results demonstrate the potential for rapid post-war recovery of large-herbivore biomass, given sound protected-area management, but also suggest that restoration of community structure takes longer and may require active intervention.

## Introduction

Ecosystems worldwide have been altered by faunal declines and extirpations, which have accelerated sharply over the past century [1,2]. Large mammalian herbivores (> 5 kg) are particularly vulnerable to anthropogenic impacts due to their extensive habitat requirements, long generation times, and human demand for their meat, hides, horns, and ivory [3–6]. Indeed, recent decades have seen global reductions of large-mammal populations, especially for species >100 kg [5]. Throughout most of Africa, the abundance of many taxa decreased by nearly 60% between 1970 and 2005 [7] due to habitat loss, climatic shifts, exploitation, and displacement by growing human populations [8,9].

Armed human conflict can also be a potent driver of wildlife declines and biodiversity loss, but its ecological impacts remain little-studied [10–13]. The majority of wars since 1950 have been fought in tropical Africa and Asia [14], home to the greatest diversity of extant large mammals. In Africa, armed conflict has generally been associated with wildlife declines of varying magnitude [12]. These declines can result from a tangle of interrelated mechanisms, including human displacement, livelihood loss, erosion of social networks and norms, relaxation or disintegration of governance, trade of animal products for weaponry, and general economic malaise [10]. At the same time, however, efforts to conserve and restore heavily impacted landscapes create opportunities to rehabilitate large-mammal assemblages following such mass-mortality events [15–17].

Successfully managing the rehabilitation of large-mammal populations ideally requires an understanding of pre-disturbance baselines, the rates and magnitudes of anthropogenic declines, and post-disturbance population trajectories [17]. In addition to informing the conservation and management of recovering large-mammal populations, studying these dynamics can inform long-standing debates in ecology about community (re)assembly, competition, species coexistence, and the degree to which communities return to pre-perturbation baseline states. Such work is particularly relevant in African savannas, where episodic large-mammal die-offs may govern ecosystem structure and function: windows of relaxed grazing or browsing pressure, for example, are thought to shape the dynamic tree-grass balance in African savannas [18–21].

Because controlled experimental manipulations of large-mammal declines and recovery at relevant spatial and temporal scales are infeasible, there is enormous value in characterizing the multi-decadal population trends of real assemblages before and after mass-mortality events. Data from well documented perturbations, while scarce, can be used to parameterize models of population dynamics and viability [20,22] and provide information on the sensitivity of populations to changing environmental conditions and the consequences of de- and re-faunation on vegetation structure, carbon storage, and ecosystem services [19,23,24]. Most long-term studies of large-mammal population trends come from countries with relatively stable histories and successful wildlife-conservation efforts, such as Kenya, Tanzania, and South Africa (e.g., [9,25–27]). Long-term records of large-mammal populations in countries with recent histories of war and instability are scarce, providing scant basis for assessing how wildlife assemblages disassemble and reassemble in the wake of conflict and other major anthropogenic perturbations.

Here, we present data from 15 aerial surveys in Mozambique’s Gorongosa National Park (GNP), spanning half a century from 1968 to 2018. GNP was once touted as one of Africa’s most spectacular national parks, with massive herds of wildlife roaming its Rift Valley grasslands and woodlands. During Mozambique’s post-colonial civil war (1977–1992), in which hundreds of thousands of people were killed, hostilities raged in and around the park. This conflict, and the poverty that persisted after the fighting ended, severely reduced the park’s large mammals. Wildlife recovery has accelerated since 2004, coinciding with the public-private co-management activities of the Gorongosa Project [28]. Our aim in this paper is to provide a systematic quantitative accounting of both the magnitude of war-induced declines and the dynamics of the ongoing recovery for the major large-herbivore species in GNP. Existing data are insufficient to enable assessment of the mechanisms underlying the patterns we identify, which are the subject of ongoing research. Rather, we seek to synthesize the available data, characterize salient trends, and suggest ideas to guide future studies.

## Materials and methods

### Study site

**Fig 1:**
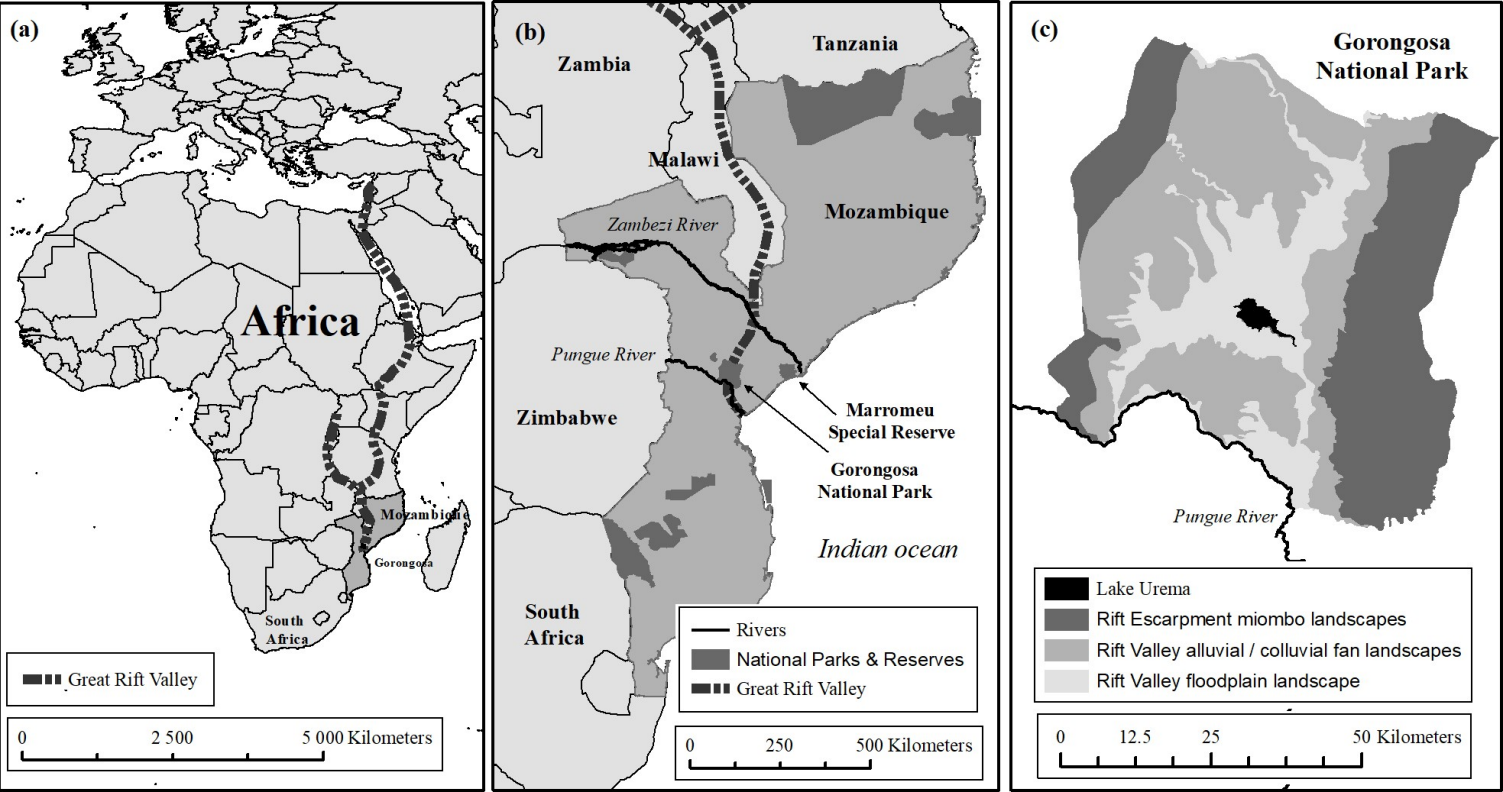
**Map of Gorongosa National Park (GNP) in Mozambique**, showing (a) Locality of the Great Rift Valley and of Mozambique within Africa; (b) Location of GNP and other Protected Areas (dark gray) within Mozambique; and (c) Major landscape types of GNP.

The GNP was proclaimed in 1960 and covers 3,674 km^2^ of Sofala Province, Mozambique, at the southernmost tip of the Great African Rift (Fig 1). Tinley [29] provided a detailed account of GNP’s geography and ecology between 1968 and 1972, before the park was heavily affected by war. The Rift Valley is the salient geological feature of the area, and the 40-km wide valley floor (15–80 m elevation) is flanked on the east and west by hilly terrain rising to 300–400 m above sea level. Mean annual rainfall within the Rift is 700–900 mm, with greater rainfall recorded on the valley sides. Large areas of the Rift Valley are seasonally inundated following the December–February peak rainfall, resulting in extensive floodplains around the central Lake Urema. Fifteen landscape types are recognized in GNP, with floodplain grasslands and *Acacia-Combretum* savanna predominating in the Rift Valley and miombo woodlands occurring at higher elevations to the east and west [30] (Fig 1C).

Historically, GNP and surrounding areas supported a wide diversity of large mammalian herbivores, which attained highest densities within the Rift Valley, particularly on the Urema floodplain. These areas were dominated by herds of buffalo (*Syncerus caffer*), blue wildebeest (*Connochaetes taurinus*), zebra (*Equus quagga*), waterbuck (*Kobus ellipsiprymnus*), and impala (*Aepyceros melampus*), with a large population of hippo (*Hippopotamus amphibius*) occurring within Lake Urema and associated rivers and troughs. Elephant (*Loxodonta africana*) were also abundant and ranged throughout the park. Other common large herbivores included kudu (*Tragelaphus strepsiceros*), nyala (*T*. *angasii*), bushbuck (*T*. *sylvaticus*), sable antelope (*Hippotragus niger*), Lichtenstein’s hartebeest (*Alcelaphus lichtensteinii*), common reedbuck (*Redunca arundinum*), oribi (*Ourebia ourebi*), and warthog (*Phacochoerus africanus*). Roan antelope (*Hippotragus equinus*), tsessebe (*Damaliscus lunatus*), and rhino (*Diceros bicornis* and *Ceratotherium simum*) were recorded in early explorers’ accounts but had been effectively extirpated by the late 1960s [29]. Top predators included lion (*Panthera leo*), leopard (*P*. *pardus*), hyena (*Crocuta crocuta*), wild dog (*Lycaon pictus*), jackal (*Canis adustus*), and crocodile (*Crocodylus niloticus*).

Mozambique’s War of Independence against Portugal ended in 1974 and was followed by the Mozambican Civil War (1977–1992). During and immediately after the civil war—which centered in Sofala province and Gorongosa specifically—GNP’s wildlife was heavily hunted for food and sale, including ivory used to finance the conflict [31]. The first post-war aerial census of GNP’s Rift Valley floor was completed in June, 1994, revealing a catastrophic decline in the abundance of all large-herbivore populations [32]. Carcasses seen on this survey were old, leading Cumming *et al*. [33] to conclude that the major declines in species such as buffalo, elephant, and hippo had taken place before 1990.

Beginning in 2004, the Carr Foundation, a US-based non-profit, initiated the Gorongosa Restoration Project—a 20-year co-management agreement with the Mozambican Government to support conservation, science, and human- and economic-development activities within the park and its surrounding 5,402 km^2^ buffer zone [28]. This agreement has since been extended through the year 2040. The Gorongosa Project aims to use science to inform adaptive management of GNP’s ecosystems and wildlife, and to harmonize conservation with the needs and aspirations of the ~200,000 people residing in the buffer zone. The restoration strategy centers predominantly on facilitating natural recovery of remnant populations by protecting the resource base, reducing illegal hunting, and engaging local communities. This natural recovery process has been complemented for some species via translocations and reintroductions from elsewhere in southern Africa (S1 Table).

### Aerial wildlife surveys

**Fig 2:**
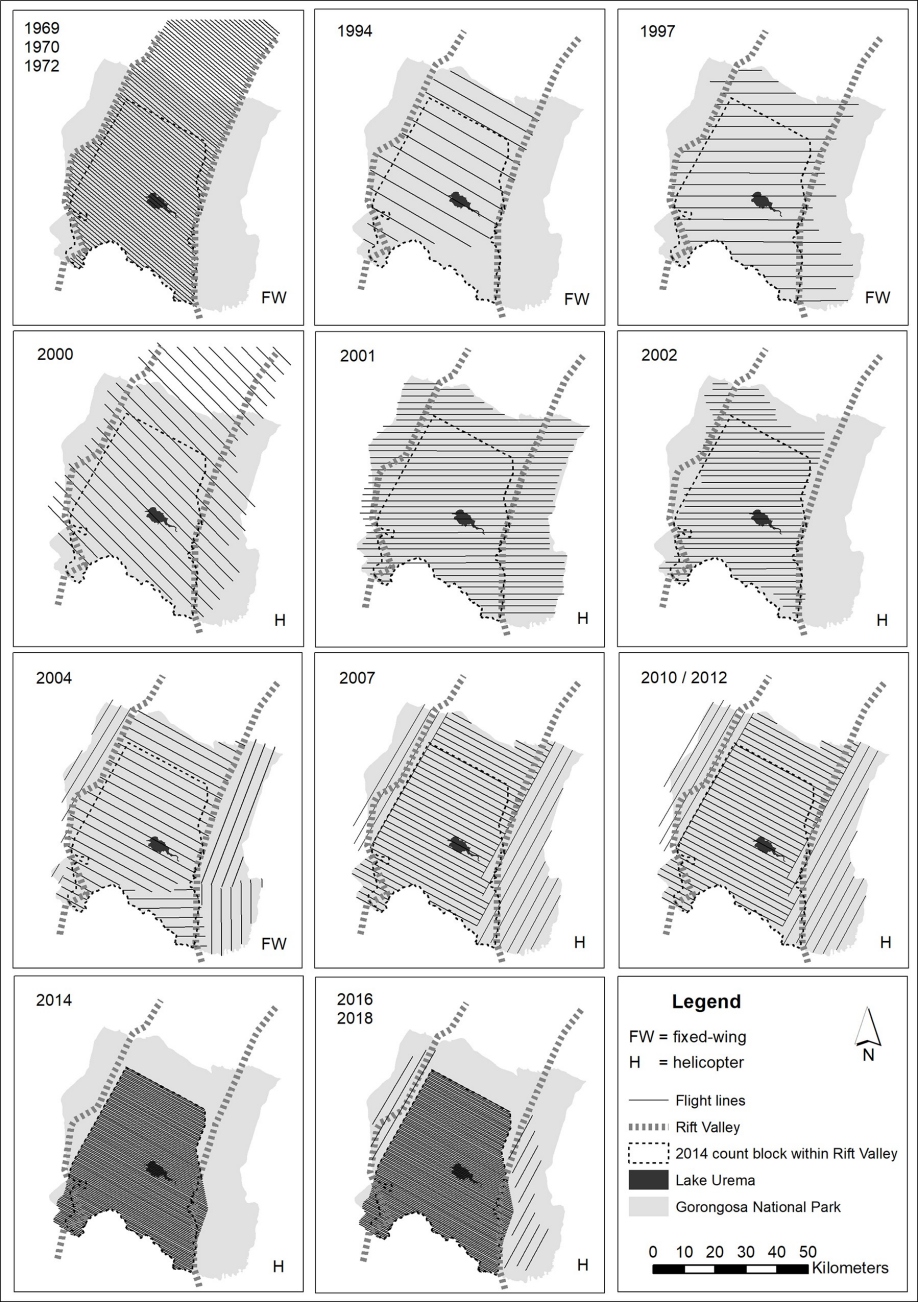
Layout of the flight strips (solid lines) in the aerial wildlife counts from 1968–2018 in GNP. Thick dashed lines indicate sides of the Rift Valley. Thin dashed line shows the boundaries of the 183,163-ha count block used from 2014–2018 (bottom panels), which was subsequently used to subset the data from all surveys, so that we only analyzed data from this common area.

Six pre-war wildlife surveys were conducted from 1968–1972, and 12 post-war surveys were conducted between 1994 and 2018. The 1968–1972, 1994, 1997, and 2004 surveys were conducted using a fixed-wing aircraft. All surveys from 2000 onwards, with the exception of 2004, were conducted via helicopter. Methodologies and coverage are outlined below and also in Table 1 and Fig 2.

**Table 1:** Summary of aerial count survey methodology (* denotes lack of georeferenced data).

Aerial count year	Month	Type of platform	Type of count	Strip width (m)	% of Rift Valley sampled	% of GNP sampled
1968 late dry season *	November	Fixed-wing	Full count	1,000	100.0	64.7
1969 wet season *	February	Fixed-wing	Full count	1,000	100.0	64.7
1969 late dry season	October	Fixed-wing	Full count	1,000	100.0	64.7
1970 wet season	January	Fixed-wing	Full count	1,000	100.0	64.7
1971 wet season *	March	Fixed-wing	Full count	1,000	100.0	64.7
1972 late dry season	October	Fixed-wing	Full count	1,000	100.0	64.7
1994 late dry season	June	Fixed-wing	Sample count	255	4.2	2.9
1997 late dry season	October	Fixed-wing	Sample count	255	6.5	5.2
2000 late dry season	October	Helicopter	Sample count	500	13.6	11.6
2001 late dry season	November	Helicopter	Sample count	500	24.8	24.4
2002 late dry season	November	Helicopter	Sample count	500	21.4	14.6
2004 late dry season	October	Fixed-wing	Sample count	303	9.9	9.6
2007 late dry season	November	Helicopter	Sample count	500	24.7	21.9
2010 late dry season	November	Helicopter	Sample count	500	24.7	22.5
2012 early dry season	May	Helicopter	Sample count	500	25.8	23.8
2014 late dry season	October	Helicopter	Full count	500	71.8	49.9
2016 late dry season	October	Helicopter	Full count with additional sample lines outside of Rift Valley	500	73.0	53.9
2018 late dry season	October	Helicopter	Full count with additional sample lines outside of Rift Valley	500	74.2	58.9

Tinley [29] conducted the six semi-annual surveys (in both wet and dry seasons) between November 1968 and October 1972. Strips 1,000-m wide (500 m on each side of the aircraft) were flown at ~150 m altitude and ~160 km/h. The entire Rift Valley was covered, representing 64.7% of GNP. Eight species were counted in all of these surveys (elephant, buffalo, wildebeest, zebra, waterbuck, eland, sable, hartebeest); hippo were counted only in October 1968 and November 1972. As full spatial information on wildlife distributions was available only for the 1969 dry-season, 1970 wet-season, and 1972 dry-season surveys, we used only those data in our analysis; for hippos, we used 1968 data for 1969 and 1970.

Two years after the cessation of hostilities, Cumming *et al*. [33] conducted the first post-war survey. Strips 255-m wide were flown at 91 m altitude and ~160 km/h, yielding 4.2% and 2.9% coverage of the Rift Valley and GNP, respectively. Three years later, Dutton [34] flew a similar survey, achieving 6.5% and 5.2% coverage of the Rift Valley and GNP, respectively. Dunham’s [35] fixed-wing survey flew 300-m wide strips at 160 km/h and 91 m altitude to achieve 9.9% and 9.6% coverage of the Rift Valley and GNP, respectively.

Sample counts were conducted via helicopter in 2000, 2001, 2002, 2007, 2010, and 2012 by flying 500-m wide strips at 150 km/h, at a radar-altimeter altitude of 60–90 m. Between 13.6 and 24.8% of the Rift Valley, and between 11.6 and 24.4% of GNP, was sampled in these helicopter surveys. During the 2000 survey, a number of additional transect lines (totaling 337 km and 16,850 hectares) were flown north of the current park boundary (Fig 2). In each count since 2004, a supplementary dedicated flight was done of the river and lake system where hippo occur, thus effectively covering the whole counting block. Hippo densities for the four counts from 2004 to 2012 were therefore calculated taking into account the full extent of the count block rather than the sample intensities achieved through the systematic counting lines.

The sample-count methodology used in surveys up through 2012 revealed several shortcomings. In particular, only small numbers of individual groups/herds of species such as elephant and buffalo were recorded across the widely separated flight lines (especially within the sparsely populated western-, northern-, and eastern-most parts of GNP), leading to some variable encounter rates and spurious density estimates, particularly in the 1990’s and early 2000’s. The survey method was therefore changed in 2014 to include full coverage of the southern and central portion of the Rift Valley (Fig 2). Similar flying specifications were used, but the 500-m wide strips were contiguous, allowing 100% coverage of a single 183,163-ha count block representing 71.8% of the Rift Valley and 49.9% of GNP, respectively (Table 1, Fig 2). In 2016 and 2018, we included an additional series of non-contiguous transects situated to the west (100 and 185 km respectively) and east (125 and 205 km) of the main total-count block. This amounted to 11,250 ha of additional coverage in 2016 and 19,500 ha in 2018, which we used both to assess wildlife densities outside of the primary count block and to provide baseline data from which to monitor the eventual repopulation of these outlying areas.

Surveys from 1994 onwards included species for which little-to-no pre-war data are available. From 2007–2018, these included a total of 19 herbivores (the nine large species listed above, plus impala, bushbuck, reedbuck, grey and red duiker, kudu, nyala, oribi, bushpig, and warthog), as well as the number of baboon (*Papio cynocephalus*) troops. All eight helicopter surveys were flown by the same pilot (M. Pingo, Sunrise Aviation) using a Bell Jet Ranger and the same custom-made census software. Both M. Stalmans and M. Peel participated in the 2010–2018 surveys. In total, our dataset comprises 70,102 large-herbivore sightings, each with associated date, time, latitude/longitude (digitized from survey maps for pre-war surveys and GPS positions for post-war surveys), species identity, and number of individuals observed (S1–S5 Tables, S1 File). Whereas animals were individually counted during the surveys, in 2014 and 2018, part of the waterbuck population was so concentrated that this approach risked inaccuracies. Therefore, photographs were taken of these large herds and the number of individuals was subsequently determined from the photographs. A similar approach was used during the pre-war counts for some of the large buffalo herds [29].

### Data Analysis

#### Converting raw count data into comparable density estimates

To increase comparability of data across years, we restricted our analysis to include only those records that were located within the 170,813-ha Rift Valley portion of the 2014–2018 count block (Fig 2), which includes the most-consistently surveyed habitat in the core of the park. For the pre-war and 2014–2018 total counts, we calculated densities of each species within this area by dividing the raw count data by the total area of the count block; for the 1994–2012 sample counts, we estimated densities by dividing the raw count data by the area sampled (i.e., the summed length×width of all flight strips) within the count block. A detailed rationale for this approach is presented in S1 Appendix.

#### Grouping of counts

To minimize the effect of sampling shortcomings and any resulting idiosyncrasies or biases in the data, the 15 counts were grouped as follows for graphical presentation: three pre-war counts from 1969 to 1972, five counts during early recovery from 1994 to 2002, four counts during the middle phase from 2004 to 2012, and three counts from 2014 to 2018. We calculated the means and standard errors of each response across the 3–5 surveys within each sampling interval.

#### Observed and modeled population trends

To evaluate how observed post-war population trajectories compared with a scenario of uninhibited recovery, we used data from the literature to parameterize simple logistic-growth models for two species. These models reflect the case in which population growth is constrained only by initial population size, intrinsic reproductive and death rates, and carrying capacity (i.e., populations are not substantially depressed by interspecific competition, predation, or disease) and therefore represent the ‘best-case’ recovery scenario for any given species. Thus, the models serve as a benchmark by which observed trends can be assessed relative to the maximum theoretically possible rate of increase (e.g., [36]). We used a simple age-structured logistic model with parameters derived from the PanTHERIA database [37], and with plausible lower-bound, intermediate, and upper-bound estimates of carrying capacity (*K*) for an ecosystem with GNP’s rainfall derived from published data on large-herbivore biomass from 31 African savannas [38] (S1 Fig). Full details of model specification and parameterization are provided in S2 Appendix. We present illustrative results for two species; waterbuck and hartebeest which demonstrate contrasting example of recovery dynamics. For waterbuck, the only species remotely approaching any estimate of *K*, we show observed data alongside logistic functions plotted using the low, intermediate, and high *K* estimates, with starting population size (*N*_0_) equal to the observed initial count. For the less-abundant hartebeest, all three *K* estimates yielded similar curves over the range of observed post-war densities; therefore, we show data alongside logistic curves using *N*_0_ equal to the initial count ± 50%, to reflect the possibility that early surveys under- or over-estimated true abundances.

## Results

### Collapse and initial recovery dynamics

**Fig 3:**
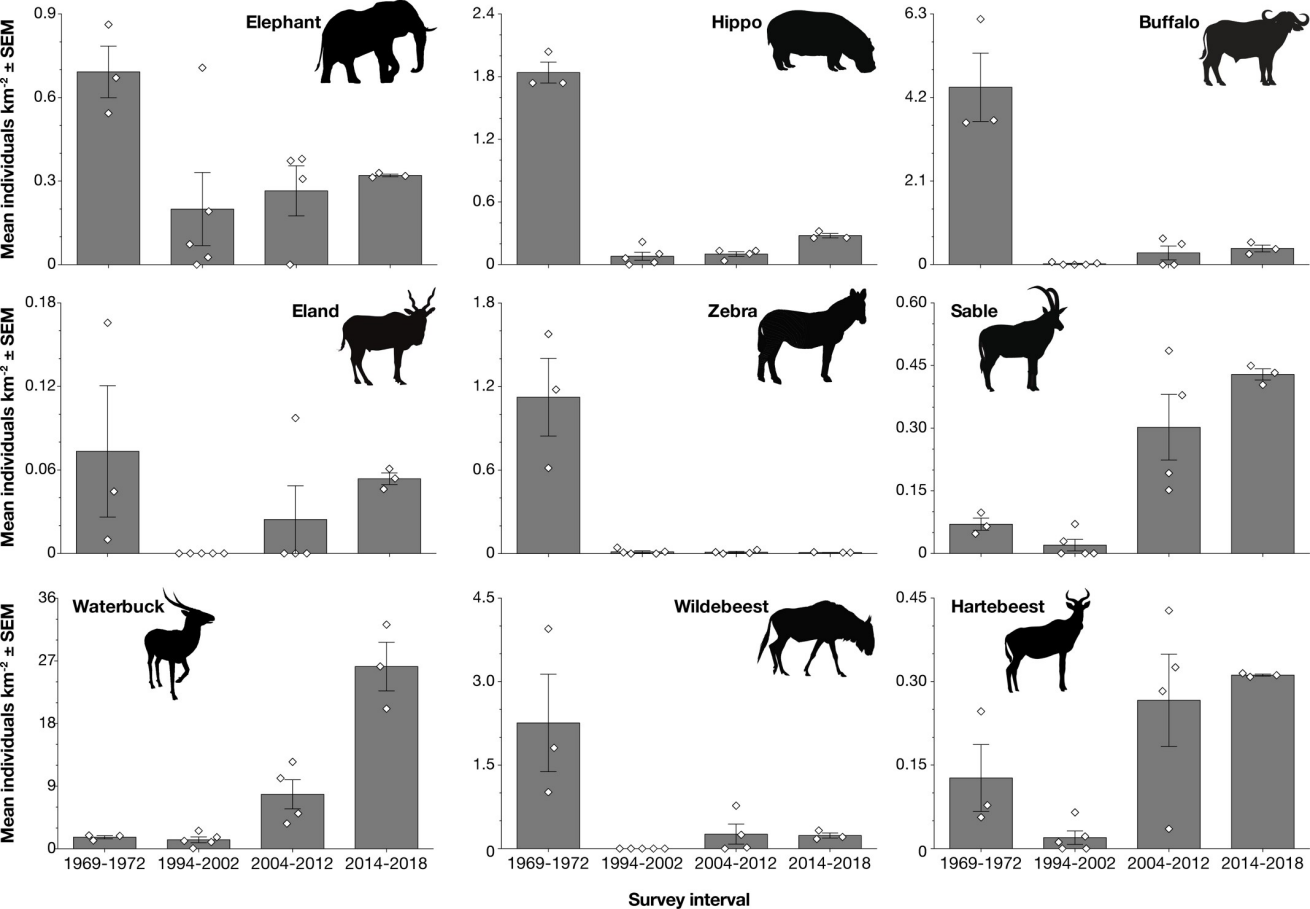
Estimated densities of nine large-bodied herbivore species through time. Bars show number of individuals per square kilometer surveyed within the core Rift Valley count block, averaged across all aerial counts within each temporal interval: pre-war surveys from 1969–1972 (n = 3), and post-war surveys in 1994–2002 (n = 5), 2004–2012 (n = 4), and 2014–2018 (n = 3). Diamonds show the values obtained for each survey within each interval. Error bars show ± 1 standard error of the mean.

Total pre-war densities averaged 12.3 individuals km^-2^ (9,298 kg km^-2^) for the nine monitored species, but declined precipitously between 1972 and early post-war surveys (Fig 3). The total estimated biomass density of these nine species, averaged across the 1994–1997 surveys, was reduced by 96% relative to the pre-war average. Likewise, the densities of elephant, zebra, and hippo declined by 93–97% over this interval (Fig 3, S2 Table). Buffalo (once the single-most abundant species) and wildebeest were not re-encountered at all until 2001 and 2007, respectively, both having declined by >99%. Small numbers of survivors from the war of both species were however observed during a number of helicopter flights that fell outside of the aerial counts.

**Fig 4:**
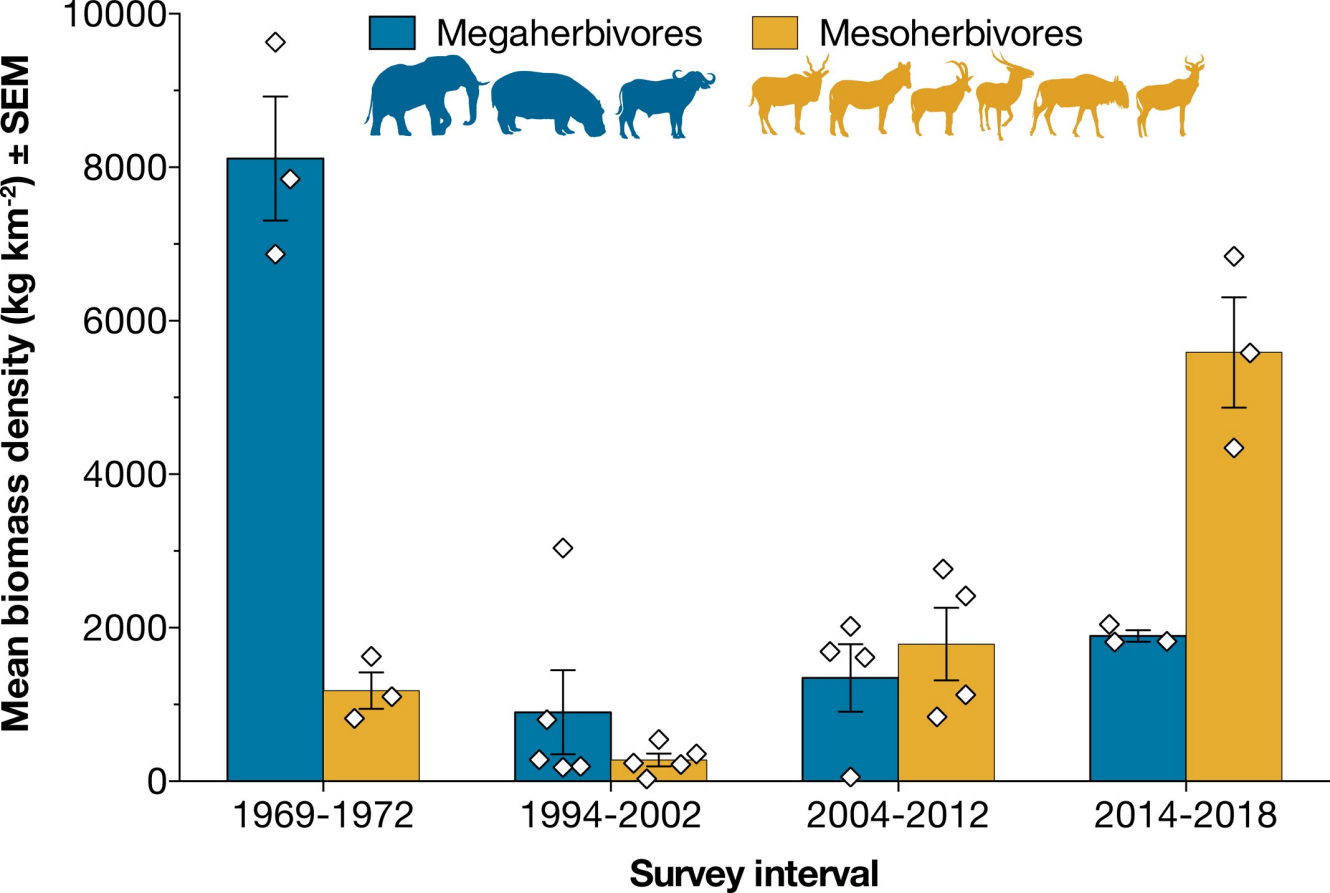
Shift in size structure of large-herbivore biomass in the core Rift Valley count block. Bars show the summed biomass densities (kilograms per square kilometer surveyed) of megaherbivores and mesoherbivores, respectively, averaged across all aerial counts within each temporal interval: pre-war surveys from 1969–1972 (n = 3), and post-war surveys in 1994–2002 (n = 5), 2004–2012 (n = 4), and 2014–2018 (n = 3). ‘Megaherbivores’ are defined here to include species with adult body mass ≥600 kg (elephant, hippo, buffalo); ‘mesoherbivores’ include the six species with body mass <600 kg (eland, zebra, sable, waterbuck, wildebeest, hartebeest) that were included in the pre-war surveys. Diamonds show the values obtained for each survey within each interval. Error bars show ± 1 standard error of the mean.

**Fig 5:**
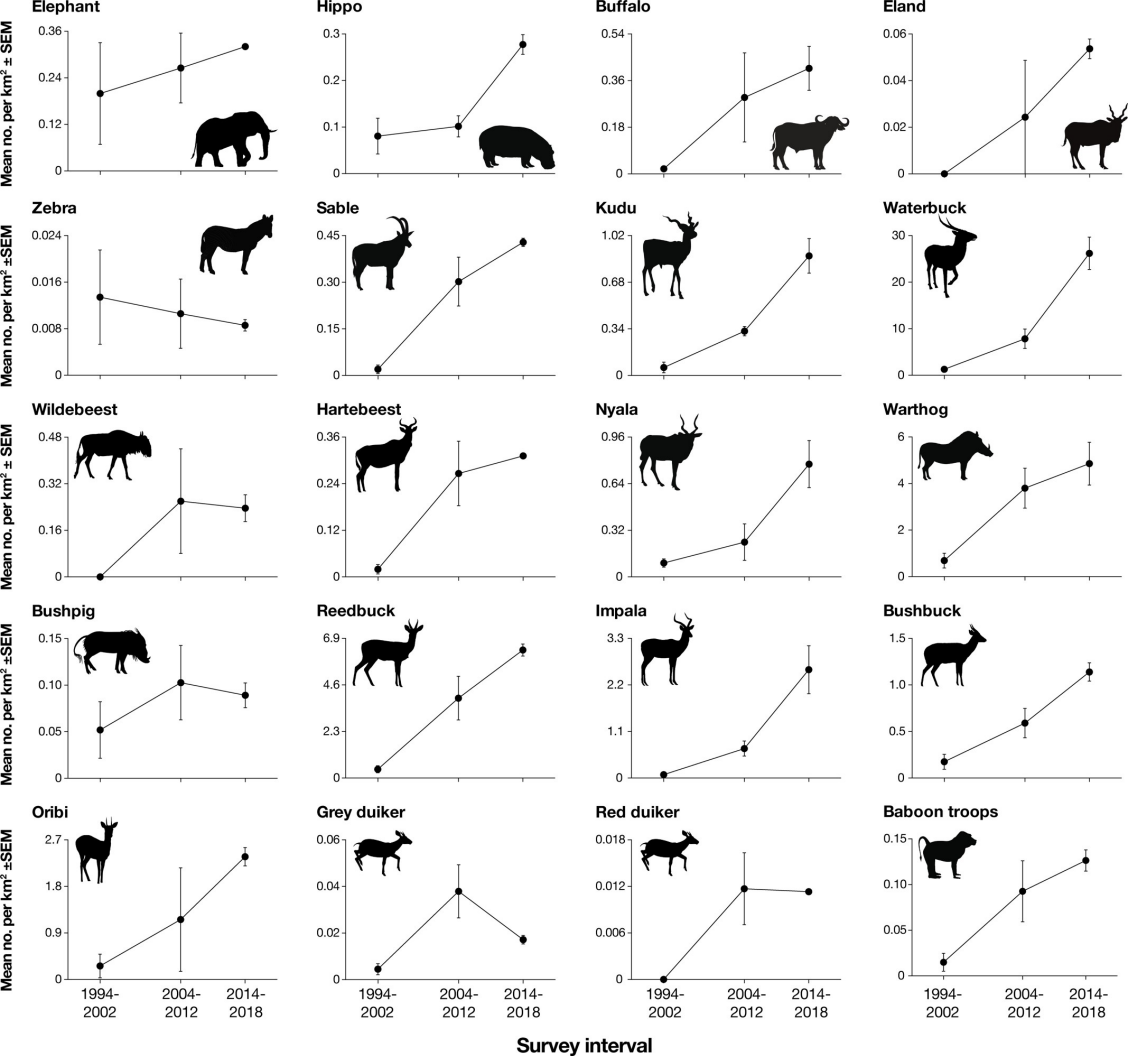
Post-war population trajectories of 20 wildlife species. Values on Y-axes are number of individuals (or troops, for baboons) per square kilometer surveyed in the core Rift Valley count block, averaged across all aerial counts within each temporal interval: 1994–2002 (n = 5 surveys), 2004–2012 (n = 4 surveys), and 2014–2018 (n = 3 surveys).

By 2010, total biomass density of these nine focal species had recovered to >50% of the pre-war baseline, and by 2018 to ~95%. However, community composition has shifted dramatically relative to the pre-war baseline owing to asymmetric recovery rates across species, with smaller antelope species supplanting the formerly dominant megaherbivores (Figs 3, 4 and 5). Most strikingly, waterbuck (200 kg), has replaced buffalo as the most dominant species in terms of both abundance and biomass. Buffalo, hippo, and elephant jointly represented >87% of mean pre-war biomass among the nine focal species, whereas waterbuck accounted for just ~4%; in contrast, waterbuck represented >74% of total biomass of these nine species by 2018 (Fig 4; S2 Table). Spatially, waterbuck have been expanding outwards from the floodplain surrounding Lake Urema and now occupy nearly the entirety of the core Rift Valley count block (S2 Fig). Estimated densities of sable and hartebeest in 2018 were also greater than those recorded by Tinley [29] before the war.

In contrast to these patterns for small to mid-sized and selectively feeding antelopes, the densities of large-bodied and bulk-feeding grazers, including hippo, buffalo, zebra, and wildebeest, remained markedly lower than pre-war levels (Figs 3, 4 and 5). Zebra in particular occurred at <2% of historic levels: in 2018, just 13 were counted within the core Rift Valley count block, although the total observed was 44 including areas outside of the Rift Valley.

Baboon density in GNP from 2007–2018 was 0.10–0.15 troops km^-2^; based on observations suggesting typical troop sizes of 30–80 individuals, this amounts to roughly 5–8 individuals km^-2^ (Fig 5). Of the large carnivores present pre-war, only the lion population persisted throughout the study interval [39]. With steadily increasing observer presence and camera-trapping effort over the past decade, individual hyena and jackal have been documented on a small number of occasions (e.g., one hyena and four jackal records from an array of 60 camera traps over 13 months from 2013 to 2014), but it remains unclear whether these are part of a viable resident population. A single male leopard was photographed in March of 2018, the first confirmed sighting in the post-war era. A pack of 14 wild dogs was reintroduced from South Africa and released into the park in June 2018. Further leopard and wild-dog translocations are planned for 2019 and onwards.

### Observed and modeled population trends

**Fig 6:**
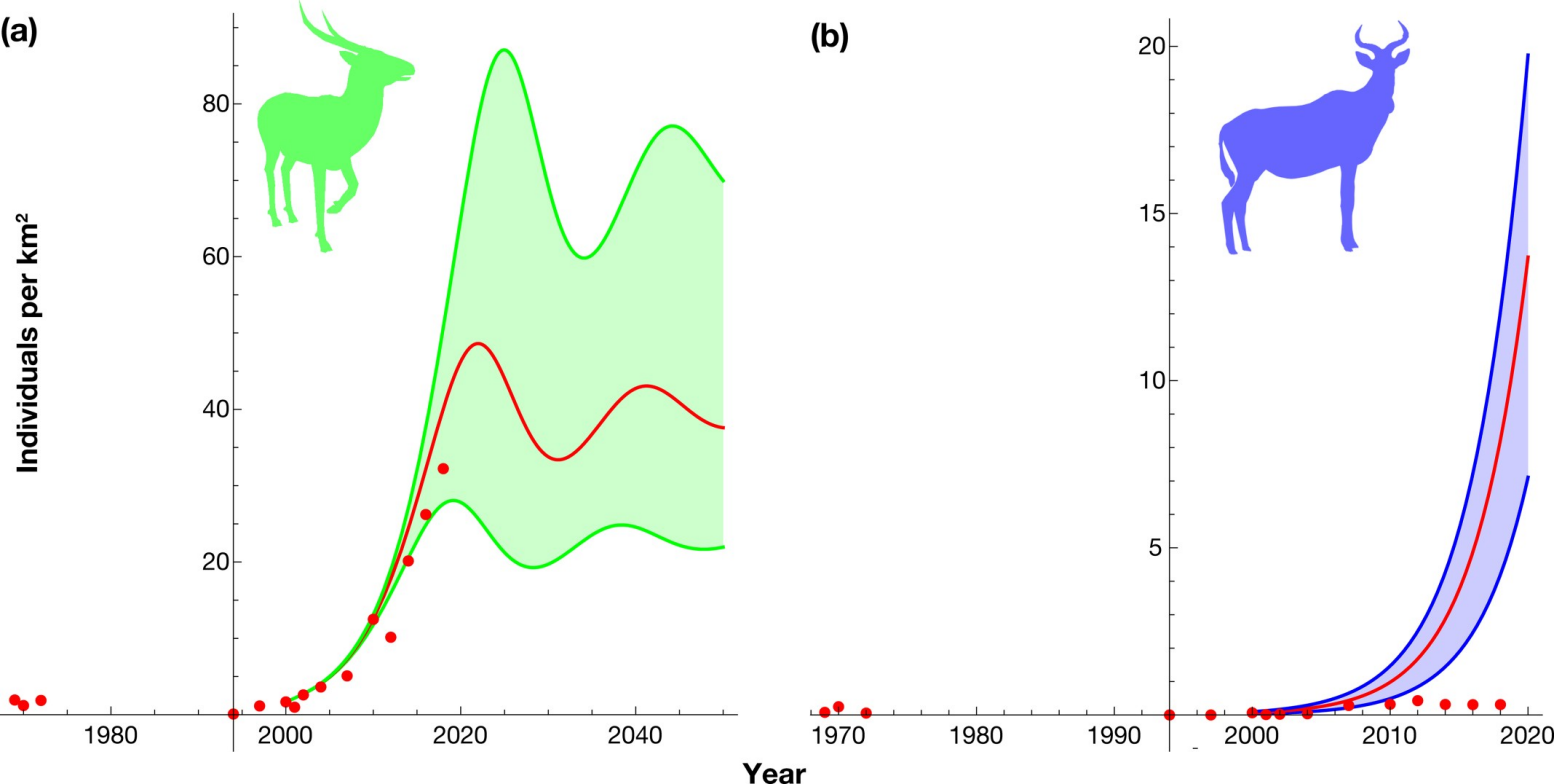
**Observed pre- and post-war aerial survey data (red points) plotted against logistic-growth curves for (a) waterbuck and, (b) hartebeest.** Vertical axes (number of individuals per km^2^ surveyed) intersects horizontal axes (year) in 1994, coinciding with the first post-war survey. For waterbuck, logistic curves are plotted using an intermediate *K* estimate (red line) along with plausible upper- and lower-bound *K* estimates (green lines and shaded interval). For hartebeest, logistic curves are plotted using the observed initial post-war density estimate (red line) ± 50% (blue lines and shaded interval), assuming the intermediate *K* value.

Comparison of observed densities with modeled logistic-growth curves suggests that the waterbuck trajectory was closely aligned with logistic expectations (Fig 6A). Hartebeest illustrate a case in which population densities fell well below the logistic prediction (Fig 6B); nonetheless, post-war hartebeest density estimates from 2007–2018 were greater than those estimated from pre-war aerial surveys (Fig 3).

## Discussion

### Overall trends

We acknowledge the potential sources of error associated with comparing count data generated using different methods, and we have tried to interpret our results with appropriate caution. In particular, results from the early post-war surveys, in which limited areal coverage combined with low abundances caused uneven encounter rates, should be regarded as approximations of uncertain precision. However, we believe that our area-corrected density estimates from within the consistently sampled Rift Valley (with its generally high visibility) are sufficient to accurately reflect the dramatic changes in relative abundance and community structure that have occurred throughout the study period.

The available data document drastic war-driven reductions in relative abundance and biomass of a large-herbivore populations in GNP, followed by variable species-specific recovery rates during the first two post-war decades. Small-to-medium-sized and selectively feeding antelopes have rapidly regained or exceeded their estimated pre-war densities, while larger-bodied and bulk-feeding species remain at a fraction of pre-war levels. We conclude that whereas total large-herbivore biomass is resilient and rebounds rapidly following catastrophic perturbation, the relative abundance of different species and trophic guilds are far slower to recover—and may not recover at all in the absence of translocations, due to critically low population size in severely affected species such as zebra (and carnivores). Although differences in the counting methods used over time affect the precision of our estimates, the large magnitude of the differences observed, and the generally high year-to-year consistency of density estimates for most species, give us confidence that our characterization is broadly accurate.

Pre-war wildlife densities in GNP were high relative to other protected areas throughout the continent. The mean estimated biomass density of >9,000 kg km^-2^ for just the nine major species surveyed by Tinley [29] (S1 Fig, S2 Table) puts GNP on par with contemporaneous estimates of large-herbivore biomass in ecosystems with famously high wildlife abundance, such as Ngorongoro Crater (>7500 kg km^-2^) and Serengeti National Park (>8300 kg km^-2^) [38]. The subsequent population declines of 90–99% from the mid-1970s through the late 1990s were severe for all nine species monitored. This conclusion is reinforced by anecdotal reports from observers familiar with pre-war conditions [32], which convey a near-total elimination of wildlife, including many smaller species not counted during the pre-war fixed-wing surveys (e.g., impala, reedbuck, bushbuck, oribi, warthog; Fig 5).

Since 2004, there has been a marked recovery of total wildlife biomass and of most individual populations, coinciding with the re-building of park infrastructure, resumption of law enforcement, and initiation of human-development programs under the Gorongosa Project. The observed increase of total community biomass largely reflects natural population growth of remnant populations. A series of wildlife reintroductions, involving seven species, took place between 2007 and 2018 (see full list in S1 Table), but the numbers were substantial only for buffalo (210 translocated from 2007–2011) and wildebeest (180 translocated in 2007). As of 2018, these two species represented just <2% of total individuals and <4% of total biomass (Figs 5 and 6). No translocations were made for any of the nine species that are currently most numerous (waterbuck, reedbuck, warthog, impala, oribi, bushbuck, kudu, nyala, and sable), which together comprised 98% of individuals and 80% of large-herbivore biomass in 2018.

Immigration of wildlife from outside of the park boundaries is unlikely to have contributed much to recovery of numbers and biomass within the park. All anecdotal information and data from both systematic and opportunistic overflights of areas surrounding GNP indicate that densities of medium-sized antelopes are very low—and larger species effectively absent—outside park boundaries, where hunting is intense and much land has been converted to agriculture. During the 2000 survey, when 337 km of transects were flown north of the current park boundary, a single sable antelope was the only individual of the nine largest-bodied herbivore species observed outside GNP. Even in the outlying areas within the park boundaries, wildlife densities remain significantly lower than those within the core count block. The total biomass recorded in 2016 and 2018 along the western- and eastern-most sample lines was only 461 and 754 kg km^-2^, respectively, relative to 8,880 kg km^-2^ within the core count block.

Although we tried to maximize comparability of data across years and are confident in the qualitative accuracy of our conclusions, we acknowledge the potential for imprecision and error associated with the divergent count methodologies utilized across years. This was unavoidable given the nature of the data available. The pre-war counts were conducted using a fixed-wing aircraft with wide counting strips. Undercounts of animals in the Kruger National Park, using a fixed-wing plane and a strip width of 800 m, were estimated at 15% for easily spotted species such as wildebeest and zebra and 40% for less-visible animals like impala and waterbuck [40]. However, because the pre- vs. post-war comparisons focused exclusively on nine relatively large species, all within Rift Valley habitat with relatively high and spatially homogeneous visibility, we do not think that this source of error unduly compromised our results; in any event, the effect of such error would be to reduce pre-war estimates relative to post-war ones, making our results a conservative indicator of the magnitude of war-induced declines. Post-war counts used much narrower counting strips, and since 2000 (except 2004) have been conducted by helicopter, which yields more accurate results [41]. The probability of sample counts to encounter the small number of remnant animals—especially the very limited numbers of herd-forming species—remained low until at least 2007. Thus, the precision of estimates from the 1994–2004 counts should be regarded cautiously (as reflected in our decisions to average across surveys within broad temporal intervals in Figs 3, 4 and 5). It is clear from ground-based and opportunistic aerial observations that species such as buffalo, wildebeest, and elephant did survive the war in small numbers, yet they were not picked up in some of the earliest post-war sample counts. This problem is illustrated by the improbably high elephant density estimated in 2000, reflecting the chance encounter of the bulk of a clumped population despite small sampling coverage; in contrast, no elephants were recorded in the subsequent survey in 2002, despite nearly doubling the sampling area.

### Observed and modeled population trends

Use of simple logistic models as a best-case theoretical benchmark enables us to assess both the plausibility of density estimates inferred from count data and the extent to which population-recovery rates are constrained by intrinsic (gestation length, calving interval, weaning age) vs. extrinsic (poaching, predation, disease) factors. Waterbuck densities corresponded closely with predictions of a logistic model constructed using waterbuck life-history parameters obtained from the literature. This suggests that population growth of GNP’s most abundant herbivore species has as yet been unhindered by interspecific competition, predation, poaching, or disease, and was constrained only by intraspecific density dependence (starting in around 2010). By contrast, hartebeest populations have not grown logistically, but nonetheless exceed pre-war hartebeest densities, suggesting that this species might have been constrained by extrinsic factors in both the pre- and post-war eras.

Predation is as yet unlikely to be among the extrinsic factors constraining overall herbivore recovery. In 2016, lion densities were < 30% of those that could theoretically be supported by the available prey biomass [39], and leopard and wild dog were not observed until 2018. The degradation of the large-carnivore assemblage may be one factor contributing to the rapid population growth of small-to-mid-sized ungulates, which tend to be predator-limited in more-intact African ecosystems [42]. Snaring by humans remained frequent through 2014, especially in the outlying parts of the park (which may partly explain the much lower recorded wildlife densities in these areas), but its impact on wildlife population trajectories has not been formally evaluated. Following a complete re-structuring and re-training of law-enforcement personnel in the Park, preliminary data indicate a 49% decline in snaring pressure (snares and traps) between 2016 and 2017 [39] Between 2015 and 2016, Park rangers confiscated 12363 snares and 317 steel-jaw traps from the across the Park [39]

At present, we are unable to explain why waterbuck have emerged as the overwhelmingly dominant post-war species. One likely possibility is that waterbuck survived the war in relatively higher numbers (i.e., greater N_0_); although this is not evident in the first post-war survey in 1994, it is consistent with the higher numbers of waterbuck relative to other species from 1997–2001 (Figs 3, 5 and 6; S2–S4 Tables). Waterbuck may have survived in larger numbers because they can occur year-round in floodplain grasslands, which is unique among GNP ungulate species [29]. This habitat is inaccessible by humans for much of the year, direct approach is visible from a great distance, and there are few-if-any trees or other natural features with which to set snares. Thus, a combination of higher residual population numbers and greater reproductive rate could explain the much greater abundance and biomass of waterbuck relative to the pre-war dominant species, buffalo, hippo and elephant.

The asymmetric post-war resurgence of different species raises the prospect of a persistent alternative community structure in which the most rapidly recovering species competitively suppress other populations. Recent analysis of herbivore diet composition in GNP showed that waterbuck have an extremely broad dietary niche that overlaps substantially with those of almost all other ungulates [43] indicating the potential for such competition, although the existence of competitive effects has not yet been demonstrated. Thus it remains to be seen whether the current community is a transient step en route to something resembling the pre-war state—as occurred following the creation of Kenya’s Lake Nakuru National Park, where rapid growth of waterbuck and warthog populations from 1970–1980 was later reversed as zebra, buffalo, and other species increased [27]—or rather an alternative state that will persist in the absence of active intervention.

## Conclusions and future directions

Our study demonstrates the crucial role of regular wildlife censuses for monitoring the reassembly of war-impacted African ecosystems. Future modeling and empirical work should incorporate our growing understanding of ungulate diet composition [43] and vegetation dynamics [21] in GNP to parameterize multispecies models that might help to forecast population trajectories as large-herbivore numbers continue to increase and interspecific competition intensifies. In addition, there is a need to explore the impact of predation on population trajectories, ideally informed by better data on the distribution and intensity of human hunting pressure in GNP and by analyses of both the consumptive and non-consumptive effects of carnivores [42,44,45]. The importance of apex predators in governing prey populations and species coexistence in diverse ecosystems worldwide [23,46] provides grounds to hypothesize that reestablishment of the large-carnivore community may be an essential step in the continuing ecological rehabilitation of the Gorongosa ecosystem.

## Supporting information

S1 Appendix Converting raw count data into comparable figures. (DOCX)

S2 Appendix Construction of the logistic-regression model. (DOCX)

S1 Fig Estimation of total large-herbivore carrying capacity (*K*_*tot*_). Data are from [38]. Blue lines show 90^th^ and 10^th^ quantile regression lines, used as estimates of upper and lower plausible estimates of *K*_*tot*_, respectively; red line shows ordinary least squares regression. Thin gray lines show estimates of mean annual precipitation (vertical) and biomass density (horizontal) in Gorongosa for the nine large herbivore species surveyed from 1969–1972 [29].
(DOCX)

S2 Fig Spatial pattern of waterbuck sightings based on spatially equivalent flight lines in helicopter surveys of the Rift Valley portion of Gorongosa from 2001 to 2016. For 2014 and 2016, only data from the same sample lines as used in earlier counts are included, and all years are clipped to show only the spatial extent of the 2014/2016 ‘total counts.’ A sighting consists of a single discrete observation of one or more animals. Note the expanding trend outwards from the core distribution area that was originally centered north of Lake Urema.
(DOCX)

S1 Table Wildlife introduction and translocations into GNP. Table includes numbers of individuals, years, and localities of origin for each of seven wildlife species introduced into the park between 2007–2018 (precise sex ratios for each group are not known). Only elephant bulls were translocated in 2008. Coutada 9 is a hunting concession located ~180 km northwest of GNP.
(DOCX)

S2 Table Estimated numerical densities (individuals km^-2^) of all animals counted during aerial surveys of GNP. Rrecords are limited to Rift Valley habitat within the 2014–2016 count block. Grey cells are years in which a given species was not surveyed. These are the values used in our primary analyses in the main text. Numbers can be converted into biomass by multiplying by the species-specific body mass estimates shown in the 2^nd^ column [37].
(DOCX)

S3 Table Absolute total numbers of all animals counted during the aerial surveys of GNP and limited areas north of the park boundary (uncorrected for area and habitat covered). Grey cells are years in which a given species was not surveyed. Hatched cells are years for which no spatial information is available. [37].
(DOCX)

S4 Table Absolute total numbers of all animals counted during aerial surveys of Gorongosa National Park. Records are limited to Rift Valley habitat within the limits of the 2014–2016 count block. Grey cells are years in which given species were not surveyed. Numbers can be converted into biomass by multiplying by the species-specific body mass estimates shown in the 2^nd^ column.
(DOCX)

S5 Table Illustrative metadata for the individual sightings of wildlife in Gorongosa National Park from aerial surveys spanning the period 1969–2018. The table shows five records from the dataset; the interpretation of each column heading is described below. The full dataset of 70,102 spatially referenced sighting records is available as a supplementary online file (comma-separated values format) in **S1 File**. Readers should contact the corresponding author for additional information.
(DOCX)

S1 File Data set of 70,102 spatially referenced sighting records of wildlife from 15 aerial wildlife counts 1969–2018. (XLSX)
