# Supplementary material for: War-induced collapse and asymmetric recovery of large-mammal populations in Gorongosa National Park, Mozambique
Source: PLoS One. 2019 Mar 13;14(3):e0212864. doi: 10.1371/journal.pone.0212864 (PMC6415879; doi:10.1371/journal.pone.0212864)
Supplement: S1 Appendix — (DOCX) [file pone.0212864.s001.docx]

**S1 Appendix. Converting raw count data into comparable figures**

To increase comparability of data across years, we restricted our analysis to records located within the 170,813-ha Rift Valley portion of the 183,163-ha total 2014-2018 count block (Fig 2). For the pre-war and 2014-2018 total counts, we calculated densities of each species; for the 1994–2012 sample counts, we estimated densities by dividing count data by the area sampled. Detailed rationale for this approach is as follows.

First, all surveys covered a substantial portion of the Rift Valley in the center of GNP, whereas the outlying areas were covered at a lower intensity or not at all. The Rift Valley also has the highest visibility and greatest wildlife abundance (<7% of observations in the counts from 2000–2012 were recorded outside the Rift Valley). Further, although the Tinley surveys (1969–1972) extended north within the Rift Valley beyond the park boundaries (Fig. 2), these areas have since become more densely settled and cultivated, and large wildlife has been essentially extirpated. The retained records within the 170,813 ha of Rift Valley represent 92.4% of total records and 89.1% of all individual animals counted across the 15 surveys from 1969 to 2018 analyzed here.

Moreover, small numbers of individuals (or groups) were encountered for many species, especially during the initial post-war surveys. The maximum number of groups of animals of a specific species recorded within the Rift Valley during any one of the six surveys from 1994–2004 were: zero wildebeest, one buffalo, zero eland, six elephant, three hartebeest, 10 sable, and one group of zebra. For this reason, we were unable to generate distance-based estimates of absolute population abundances/densities, which require greater numbers of groups (e.g., [1]). Densities of each species were converted to biomass densities using published body masses typical for each species [2].

We acknowledge the potential sources of error associated with comparing count data generated using different methods, and we have striven to interpret our results with appropriate caution. In particular, results from the early post-war surveys, in which limited aerial coverage combined with low abundances caused uneven encounter rates, should be regarded as first approximations of uncertain precision. However, we believe that our density estimates from within the Rift Valley block (with its generally high visibility) are sufficient to accurately reflect the dramatic changes in relative abundance and community structure that have occurred throughout the study period.

**References**

1. Buckland ST, Anderson DR, Burnham KP, Laake JL, Borchers DL, Thomas L. Introduction to Distance Sampling. Oxford: Oxford University Press; 2001.

2. Jones, KE, Bielby, J, Cardillo M, Fritz SA, O'Dell J, Orme CDL, et al. PanTHERIA: a species‐level database of life history, ecology, and geography of extant and recently extinct mammals. Ecology 2009;90: 2648.
