# Supplementary material for: War-induced collapse and asymmetric recovery of large-mammal populations in Gorongosa National Park, Mozambique
Source: PLoS One. 2019 Mar 13;14(3):e0212864. doi: 10.1371/journal.pone.0212864 (PMC6415879; doi:10.1371/journal.pone.0212864)
Supplement: S2 Appendix — (DOCX) [file pone.0212864.s002.docx]

**S2 Appendix. Construction of the logistic-growth model**

We used a simple age-structured logistic model of the form

$$\frac{dJ}{dt}=rA-mJ\left( 1-\frac{A+J}{K} \right)-d_{J}J$$

$$\frac{dA}{dt}=mJ\left( 1-\frac{A+J}{K} \right)-d_{A}A$$

where *J* and *A* are the abundances of juveniles and adults, respectively; *r* is the reproductive rate of adults; *m* is the rate at which juveniles become sexually-mature adults; *d_J_* and *d_A_* are the respective death rates of juveniles and adults; and *K* is the estimated carrying capacity of large-herbivore biomass in the system (see below). We assumed that density has an effect on maturation, such that juveniles recruit into adults at a rate that decreases as the population approaches *K*. We obtained species-specific data on body masses, age at sexual maturity, interbirth interval, and ‘litter’ size from the PanTHERIA database [1]. Reproductive rates (number of offspring per female per year) were estimated from interbirth interval and litter size; because our model does not distinguish between males and females, we divided this number by 2 to estimate the reproductive rate (*r*) per adult individual per year, assuming 50:50 sex ratios. Death rates (*d*) were defined as the reciprocal of species’ average lifespan in captivity (e.g., waterbuck live 19 years, so per capita annual death rate *d_A_* = 1/19 = 0.053). We used captive lifespans in lieu of published field estimates because predation is currently low and resources abundant in GNP, so individuals should survive to old age. We assumed equivalent death rates for adults and juveniles (*d_J_* = *d_A_*). Maturation rates (*m*) were calculated as the reciprocal of species’ age at sexual maturity. For the initial population size (*N*_0_) of each species, we used values from the first post-war helicopter count (because helicopter counts are generally more accurate than fixed-wing counts: [2]) in which at least one individual was recorded (year 2000 for waterbuck and hartebeest). Logistic-growth models are sensitive to variation in *N*_0_, and count data can be biased upwards or downwards at very low population densities due to stochasticity in encounter rates; to account for the possibility of under- or over-counting, we fit models for hartebeests using (1) the observed initial-count value and (2) this value ± 50%.

Empirically estimating carrying capacity is challenging for any ecosystem and is therefore a source of uncertainty in such models. We derived three plausible estimates of the total large mammal herbivore carrying capacity, *K_tot_*, using data from [3] on large-herbivore biomass as a function of rainfall in 31 landscapes throughout eastern and southern Africa. We plotted biomass data against rainfall (both log-transformed) and fit an ordinary least-squares regression model (*r*^2^=0.77, *F*_1,29_=97.07, P<<0.0001) along with 10^th^ and 90^th^ quantile regressions in R using the *quantreg* package [4]. We used the fitted values from these regressions at a rainfall of 840 mm year^-1^ [5] to represent low, intermediate, and high estimates of *K_tot_*, respectively (Fig. S1). This yielded *K_tot_* estimates of 4790, 8310, and 16643 kg km^-2^. The intermediate estimate here is similar to that derived from summing the biomass-density estimates for all species in Tinley’s [5] pre-war counts from 1969, 1970, and 1972 for the subset of Rift Valley habitat circumscribed by the 2014–2018 count block (9,298 kg km^-2^), suggesting that our range of *K_tot_* plausibly encompasses the true maximum density of large herbivores in this ecosystem. Given *K_tot_*, we estimated the potential numerical carrying capacity (*K*) for each individual species by dividing *K_tot_* by the adult body mass of that species; thus, intermediate carrying capacity for waterbuck (204.4 kg) is *K* = 8310/204.4 = 40.66.

**References**

1. Jones, KE, Bielby, J, Cardillo M, Fritz SA, O'Dell J, Orme CDL, et al. PanTHERIA: a species‐level database of life history, ecology, and geography of extant and recently extinct mammals. Ecology 2009;90: 2648.

2. Bothma J. du P., Peel M.J.S., Pettit S. & Grossman D. (1990) Evaluating the accuracy of some commonly used game-counting methods. *South African Journal of Wildlife Research* 20:26-32.

3. Coe MJ, Cumming DH & Phillipson J (1976). Biomass and production of large African herbivores in relation to rainfall and primary production. *Oecologia* 22:341-354.

4. Koenker R (2016). Package ‘quantreg.’ R package version 5.29.

5. Tinley KL (1977) Framework of the Gorongosa Ecosystem. Ph.D. thesis. University of Pretoria, South Africa.
