# Supplementary material for: War-induced collapse and asymmetric recovery of large-mammal populations in Gorongosa National Park, Mozambique
Source: PLoS One. 2019 Mar 13;14(3):e0212864. doi: 10.1371/journal.pone.0212864 (PMC6415879; doi:10.1371/journal.pone.0212864)
Supplement: S1 Fig — Data are from [38]. Blue lines show 90th and 10th quantile regression lines, used as estimates of upper and lower plausible estimates of Ktot, respectively; red line shows ordinary least squares regression. Thin gray lines show estimates of mean annual precipitation (vertical) and biomass density (horizontal) in Gorongosa for the nine large herbivore species surveyed from 1969–1972 [29]. (DOCX) [file pone.0212864.s003.docx]

**S1 Fig. Estimation of total large-herbivore carrying capacity (*K_tot_*).** Data are from Tinley [1]. Blue lines show 90^th^ and 10^th^ quantile regression lines, used as estimates of upper and lower plausible estimates of *K_tot_*, respectively; red line shows ordinary least squares regression. Thin gray lines show estimates of mean annual precipitation (vertical) and biomass density (horizontal) in Gorongosa for the nine large herbivore species surveyed from 1969–1972 [2].


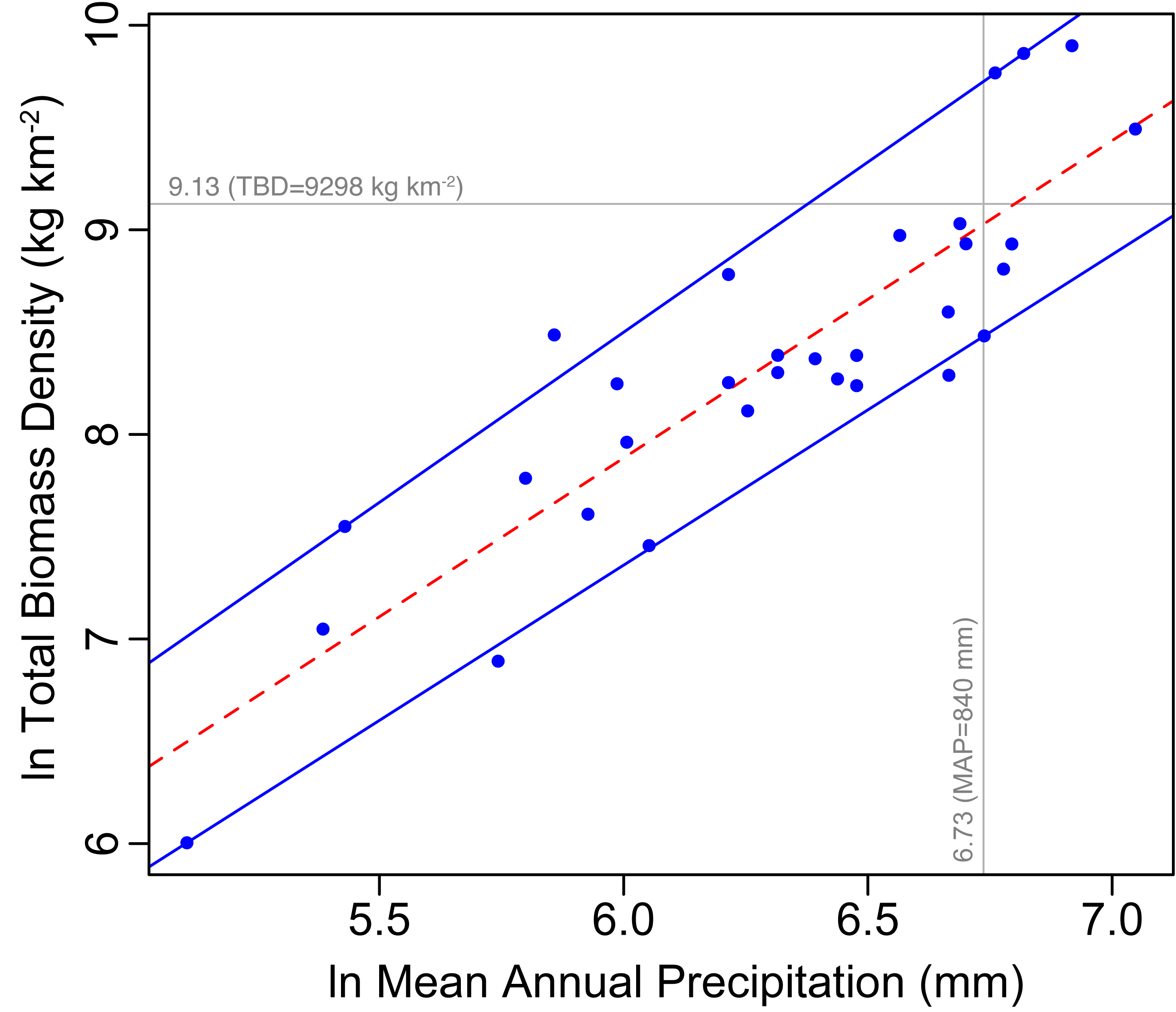


**References**

1. MJ, Cumming DH & Phillipson J (1976). Biomass and production of large African herbivores in relation to rainfall and primary production. *Oecologia* 22:341-354.
2. Tinley KL (1977) Framework of the Gorongosa Ecosystem. Ph.D. thesis. University of Pretoria, South Africa.
