# Supplementary material for: War-induced collapse and asymmetric recovery of large-mammal populations in Gorongosa National Park, Mozambique
Source: PLoS One. 2019 Mar 13;14(3):e0212864. doi: 10.1371/journal.pone.0212864 (PMC6415879; doi:10.1371/journal.pone.0212864)

**S2 Fig.** **Spatial pattern of waterbuck sightings based on spatially equivalent flight lines in helicopter surveys of the Rift Valley portion of Gorongosa from 2001 to 2016.** For 2014 and 2016, only data from the same sample lines as used in earlier counts are included, and all years are clipped to show only the spatial extent of the 2014/2016 ‘total counts.’ A sighting consists of a single discrete observation of one or more animals. Note the expanding trend outwards from the core distribution area that was originally centered north of Lake Urema.


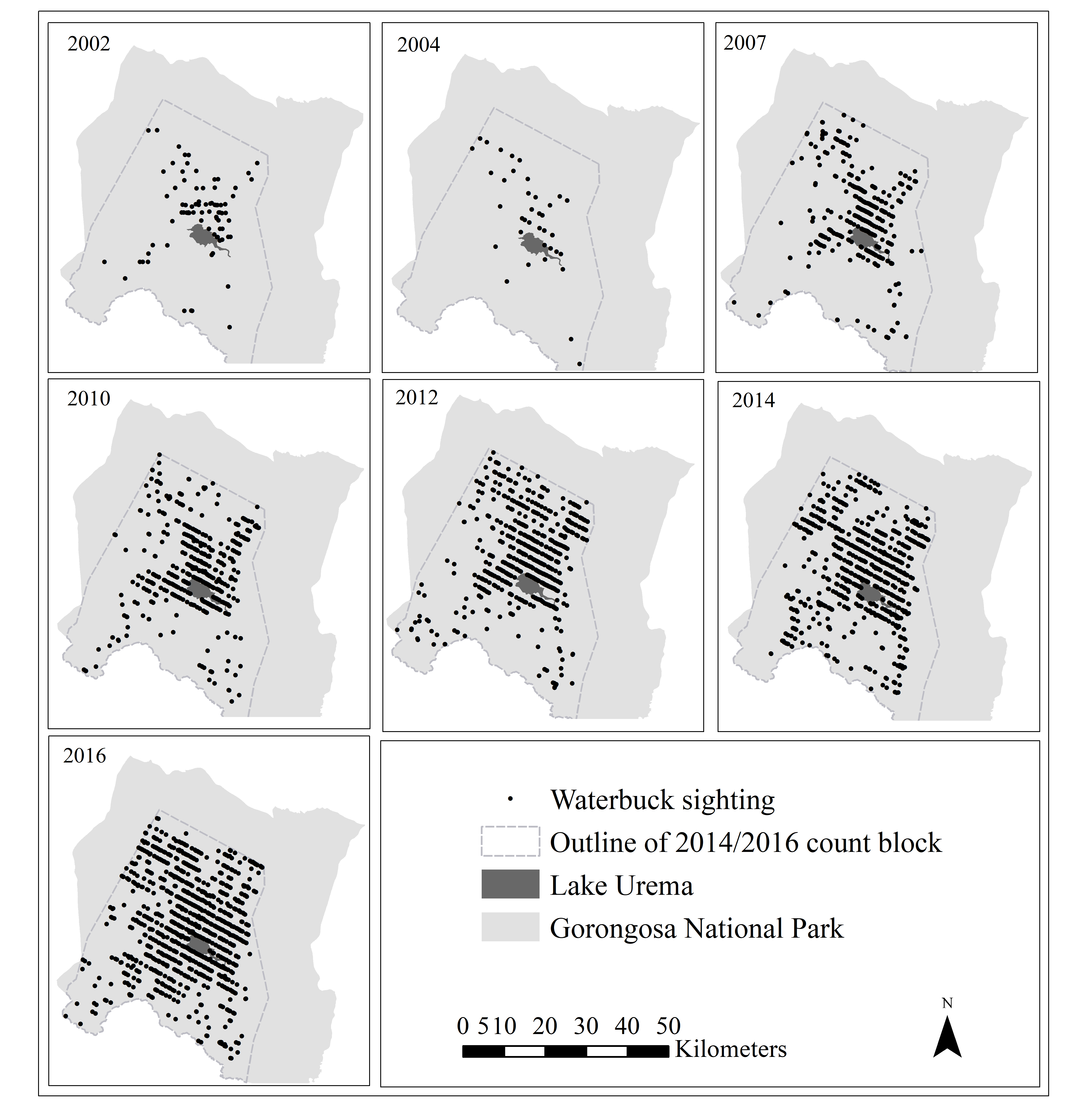

Supplement: S2 Fig — For 2014 and 2016, only data from the same sample lines as used in earlier counts are included, and all years are clipped to show only the spatial extent of the 2014/2016 ‘total counts.’ A sighting consists of a single discrete observation of one or more animals. Note the expanding trend outwards from the core distribution area that was originally centered north of Lake Urema. (DOCX) [file pone.0212864.s004.docx]
