# Supplementary material for: War-induced collapse and asymmetric recovery of large-mammal populations in Gorongosa National Park, Mozambique
Source: PLoS One. 2019 Mar 13;14(3):e0212864. doi: 10.1371/journal.pone.0212864 (PMC6415879; doi:10.1371/journal.pone.0212864)
Supplement: S1 Table — Table includes numbers of individuals, years, and localities of origin for each of seven wildlife species introduced into the park between 2007–2018 (precise sex ratios for each group are not known). Only elephant bulls were translocated in 2008. Coutada 9 is a hunting concession located ~180 km northwest of GNP. (DOCX) [file pone.0212864.s005.docx]

**S1 Table. Wildlife introduction and translocations into GNP.** Table includes numbers of individuals, years, and localities of origin for each of seven wildlife species introduced into the park between 2007–2018 (precise sex ratios for each group are not known). Only elephant bulls were translocated in 2008. Coutada 9 is a hunting concession located ~180 km northwest of GNP.

| **Species** | **Year of introduction** | **Number** | **Origin** |
| --- | --- | --- | --- |
| Blue wildebeest | 2007 | 180 | Limpopo Province, (South Africa) |
| Buffalo | 2006 | 54 | Kruger National Park (South Africa) |
| Buffalo | 2007 | 26 | Kruger National Park (South Africa) |
| Buffalo | 2007 | 31 | Limpopo National Park (Mozambique) |
| Buffalo | 2009 | 52 | Kruger National Park (South Africa) |
| Buffalo | 2011 | 47 | Kruger National Park (South Africa) |
| Eland | 2013 | 35 | Coutada 9 (Mozambique) |
| Elephant | 2008 | 6 | Kruger National Park (South Africa) |
| Hippo | 2008 | 5 | Isimangaliso Wetland Park (South Africa) |
| Zebra | 2013/2014 | 15 | Coutada 9 (Mozambique) |
| Wild dog | 2018 | 14 | Kwazulu-Natal (South Africa) |
