# Supplementary material for: War-induced collapse and asymmetric recovery of large-mammal populations in Gorongosa National Park, Mozambique
Source: PLoS One. 2019 Mar 13;14(3):e0212864. doi: 10.1371/journal.pone.0212864 (PMC6415879; doi:10.1371/journal.pone.0212864)
Supplement: S2 Table — Rrecords are limited to Rift Valley habitat within the 2014–2016 count block. Grey cells are years in which a given species was not surveyed. These are the values used in our primary analyses in the main text. Numbers can be converted into biomass by multiplying by the species-specific body mass estimates shown in the 2nd column [37]. (DOCX) [file pone.0212864.s006.docx]

**S2 Table.** **Estimated numerical densities (individuals km^-2^) of all animals counted during aerial surveys of GNP.**  Records are limited to Rift Valley habitat within the 2014-2016 count block**.** Grey cells are years in which a given species was not surveyed. These are the values used in our primary analyses in the main text. Numbers can be converted into biomass by multiplying by the species-specific body mass estimates shown in the 2^nd^ column.

|  |  |  |  |  |  | **Year of aerial count** | | |  |  |  |  |  |  |  |  |
| --- | --- | --- | --- | --- | --- | --- | --- | --- | --- | --- | --- | --- | --- | --- | --- | --- |
| **Species** | **Animal weight (kg)** | **1969** | **1970** | **1972** | **1994** | **1997** | **2000** | **2001** | **2002** | **2004** | **2007** | **2010** | **2012** | **2014** | **2016** | **2018** |
| Elephant | 3,825 | 0.544 | 0.862 | 0.671 | 0.073 | 0.026 | 0.707 |  | 0.191 |  | 0.380 | 0.373 | 0.308 | 0.313 | 0.330 | 0.318 |
| Buffalo | 593 | 3.570 | 6.173 | 3.630 |  |  |  | 0.035 | 0.063 |  | 0.002 | 0.656 | 0.522 | 0.392 | 0.269 | 0.561 |
| Wildebeest | 199 | 1.015 | 3.949 | 1.811 |  |  |  |  |  |  | 0.020 | 0.249 | 0.772 | 0.210 | 0.173 | 0.326 |
| Waterbuck | 204 | 1.927 | 1.202 | 1.870 | 0.088 | 1.147 | 1.675 | 0.980 | 2.595 | 3.632 | 5.086 | 12.496 | 10.142 | 20.144 | 26.220 | 32.227 |
| Zebra | 279 | 0.615 | 1.578 | 1.178 | 0.044 |  | 0.009 |  | 0.015 |  | 0.005 | 0.027 | 0.011 | 0.011 | 0.008 | 0.008 |
| Eland | 563 | 0.044 | 0.166 | 0.010 |  |  |  |  |  |  |  | 0.097 |  | 0.061 | 0.054 | 0.046 |
| Sable | 236 | 0.047 | 0.098 | 0.065 |  |  |  | 0.070 | 0.029 | 0.379 | 0.152 | 0.192 | 0.486 | 0.404 | 0.433 | 0.450 |
| Hartebeest | 169 | 0.078 | 0.246 | 0.056 |  |  | 0.065 | 0.012 | 0.022 | 0.036 | 0.283 | 0.319 | 0.428 | 0.315 | 0.309 | 0.311 |
| Hippo | 1,536 | 1.740 | 1.740 | 2.039 |  | 0.061 | 0.217 | 0.103 | 0.022 | 0.037 | 0.105 | 0.132 | 0.133 | 0.255 | 0.258 | 0.320 |
| Bushbuck | 43 |  |  |  |  | 0.044 | 0.365 | 0.094 | 0.373 | 0.184 | 0.801 | 0.874 | 0.503 | 1.294 | 1.168 | 0.957 |
| Bushpig | 69 |  |  |  |  |  | 0.165 | 0.059 | 0.036 |  | 0.192 | 0.124 | 0.094 | 0.097 | 0.063 | 0.107 |
| Reedbuck | 58 |  |  |  | 0.176 |  | 0.760 | 0.324 | 0.921 | 0.924 | 4.644 | 5.993 | 4.238 | 6.912 | 6.108 | 5.957 |
| Greyduiker | 16 |  |  |  |  | 0.000 | 0.013 | 0.005 | 0.005 | 0.024 | 0.043 | 0.068 | 0.017 | 0.019 | 0.019 | 0.013 |
| Impala | 53 |  |  |  |  | 0.096 | 0.139 | 0.038 | 0.131 | 0.320 | 0.573 | 0.731 | 1.170 | 1.539 | 2.643 | 3.501 |
| Kudu | 206 |  |  |  |  | 0.210 | 0.026 | 0.012 | 0.044 | 0.273 | 0.260 | 0.360 | 0.400 | 0.684 | 0.821 | 1.111 |
| Nyala | 88 |  |  |  |  | 0.140 | 0.156 | 0.061 | 0.119 | 0.077 | 0.172 | 0.611 | 0.096 | 0.517 | 0.734 | 1.072 |
| Oribi | 17 |  |  |  | 0.015 | 0.359 | 0.252 | 0.094 | 0.596 | 0.267 | 1.320 | 2.506 | 0.550 | 2.578 | 2.244 | 2.304 |
| Red duiker | 13 |  |  |  |  |  |  |  |  |  | 0.023 | 0.011 | 0.013 | 0.011 | 0.012 | 0.011 |
| Warthog | 82 |  |  |  |  | 0.342 | 0.664 | 0.582 | 1.868 | 1.422 | 3.682 | 5.133 | 4.980 | 5.233 | 3.112 | 6.225 |
