# Supplementary material for: War-induced collapse and asymmetric recovery of large-mammal populations in Gorongosa National Park, Mozambique
Source: PLoS One. 2019 Mar 13;14(3):e0212864. doi: 10.1371/journal.pone.0212864 (PMC6415879; doi:10.1371/journal.pone.0212864)
Supplement: S3 Table — Grey cells are years in which a given species was not surveyed. Hatched cells are years for which no spatial information is available. [37]. (DOCX) [file pone.0212864.s007.docx]

**S3 Table.** **Absolute total numbers of all animals counted during the aerial surveys of GNP and limited areas north of the park boundary (uncorrected for area and habitat covered).** Grey cells are years in which a given species was not surveyed. Hatched cells are years for which no spatial information is available.

|  |  |  |  |  |  |  |  |  |  | **Year of aerial count** | | | |  |  |  |  |  |  |
| --- | --- | --- | --- | --- | --- | --- | --- | --- | --- | --- | --- | --- | --- | --- | --- | --- | --- | --- | --- |
|  |  | **1968** | **1969 wet** | **1969 dry** | **1970** | **1971** | **1972** | **1994** | **1997** | **2000** | **2001** | **2002** | **2004** | **2007** | **2010** | **2012** | **2014** | **2016** | **2018** |
| **Km^2^ surveyed** | | 3,410 | 3,410 | 3,410 | 3,4100 | 3,4100 | 3,4100 | 106.0 | 191.7 | 424.6 | 896.0 | 535.1 | 347.7 | 805.5 | 827.9 | 873.2 | 1,832 | 1,944 | 2,130 |
| **Species** | **kg** |  |  |  |  |  |  |  |  |  |  |  |  |  |  |  |  |  |  |
| Elephant | 3, 825 | 1,555 | 1634 | 2,072 | 2,247 | 1,401 | 2,542 | 5 | 3 | 163 |  | 79 |  | 168 | 165 | 144 | 535 | 563 | 544 |
| Buffalo | 593 | 10,928 | 11214 | 11168 | 11 779 | 12 953 | 13,286 |  | 2 |  | 15 | 26 |  | 18 | 363 | 328 | 670 | 696 | 1,021 |
| Wildebeest | 199 | 3,020 | 2,989 | 2,734 | 7,061 | 5,618 | 6,427 |  |  |  |  |  |  | 23 | 119 | 377 | 361 | 363 | 627 |
| Waterbuck | 204 | 1,930 | 1,604 | 3,557 | 2,223 | 2,697 | 3,372 | 6 | 151 | 408 | 418 | 1,071 | 627 | 2,295 | 5,643 | 4,848 | 34507 | 45280 | 57016 |
| Zebra | 279 | 1,196 | 2,899 | 2,391 | 3,583 | 2,938 | 3,329 | 3 | 2 | 6 | 5 | 6 |  | 2 | 17 | 5 | 33 | 34 | 44 |
| Eland | 563 | 134 | 226 | 342 | 429 | 356 | 127 |  |  |  | 20 |  |  |  | 43 | 3 | 105 | 118 | 142 |
| Sable | 236 | 436 | 84 | 628 | 360 | 352 | 486 |  | 19 | 40 | 101 | 12 | 111 | 132 | 225 | 272 | 786 | 863 | 968 |
| Hartebeest | 169 | 87 | 352 | 472 | 839 | 603 | 349 |  | 7 | 36 | 6 | 9 | 7 | 166 | 195 | 252 | 613 | 569 | 647 |
| Hippo | 1,536 | 2,972 |  |  |  |  | 3 483 |  | 7 | 50 | 44 | 9 | 63 | 179 | 226 | 227 | 436 | 440 | 546 |
| Bushbuck | 43 |  |  |  |  |  |  |  | 8 | 220 | 100 | 197 | 75 | 515 | 542 | 309 | 2,294 | 2,062 | 1,787 |
| Bushpig | 69 |  |  |  |  |  |  |  | 1 | 35 | 27 | 15 | 1 | 99 | 66 | 46 | 181 | 115 | 203 |
| Reedbuck | 58 |  |  |  |  |  |  | 16 |  | 247 | 239 | 392 | 186 | 2,259 | 2,824 | 2,119 | 11912 | 10609 | 10820 |
| Grey duiker | 16 |  |  |  |  |  |  |  |  | 13 | 18 | 3 | 13 | 69 | 85 | 23 | 65 | 61 | 66 |
| Impala | 53 |  |  |  |  |  |  |  | 11 | 53 | 16 | 61 | 54 | 275 | 391 | 584 | 2 735 | 4,721 | 6,274 |
| Kudu | 206 |  |  |  |  |  |  |  | 26 | 51 | 40 | 22 | 63 | 196 | 259 | 245 | 1 223 | 1,491 | 2,105 |
| Nyala | 88 |  |  |  |  |  |  | 1 | 20 | 139 | 149 | 71 | 19 | 165 | 401 | 49 | 964 | 1,320 | 2,269 |
| Oribi | 17 |  |  |  |  |  |  | 1 | 50 | 116 | 87 | 306 | 60 | 631 | 1 207 | 272 | 4 490 | 3,896 | 4,027 |
| Red duiker | 13 |  |  |  |  |  |  |  |  |  |  |  |  | 46 | 31 | 10 | 27 | 22 | 28 |
| Warthog | 82 |  |  |  |  |  |  |  | 40 | 274 | 320 | 801 | 279 | 1 747 | 2 467 | 2 511 | 9 158 | 5 400 | 11274 |
