# Supplementary material for: War-induced collapse and asymmetric recovery of large-mammal populations in Gorongosa National Park, Mozambique
Source: PLoS One. 2019 Mar 13;14(3):e0212864. doi: 10.1371/journal.pone.0212864 (PMC6415879; doi:10.1371/journal.pone.0212864)
Supplement: S4 Table — Records are limited to Rift Valley habitat within the limits of the 2014–2016 count block. Grey cells are years in which given species were not surveyed. Numbers can be converted into biomass by multiplying by the species-specific body mass estimates shown in the 2nd column. (DOCX) [file pone.0212864.s008.docx]

**S4 Table.** **Absolute total numbers of all animals counted during aerial surveys of Gorongosa National Park.** Records are limited to Rift Valley habitat within the limits of the 2014-2016 count block. Grey cells are years in which given species were not surveyed. Numbers can be converted into biomass by multiplying by the species-specific body mass estimates shown in the 2^nd^ column.

|  |  |  |  |  |  |  | **Year of aerial count** | | |  |  |  |  |  |  |  |
| --- | --- | --- | --- | --- | --- | --- | --- | --- | --- | --- | --- | --- | --- | --- | --- | --- |
|  |  | **1969** | **1970** | **1972** | **1994** | **1997** | **2000** | **2001** | **2002** | **2004** | **2007** | **2010** | **2012** | **2014** | **2016** | **2018** |
| **Km^2^ surveyed** | | 1,708 | 1,708 | 1,708 | 68 | 114 | 230 | 426 | 413 | 169 | 442 | 442 | 467 | 1,708 | 1,708 | 1,708 |
| **Species** | **kg** |  |  |  |  |  |  |  |  |  |  |  |  |  |  |  |
| Elephant | 3,825 | 929 | 1,473 | 1,146 | 5 | 3 | 163 |  | 79 |  | 168 | 165 | 144 | 535 | 563 | 544 |
| Buffalo | 593 | 6,098 | 10,545 | 6,200 |  |  |  | 15 | 26 |  | 1 | 290 | 244 | 670 | 697 | 959 |
| Wildebeest | 199 | 1,734 | 6,745 | 3,093 |  |  |  |  |  |  | 9 | 110 | 361 | 358 | 363 | 556 |
| Waterbuck | 204 | 3,291 | 2,054 | 3,194 | 6 | 131 | 386 | 418 | 1,071 | 613 | 2,247 | 5,521 | 4,741 | 34,409 | 44,787 | 55,048 |
| Zebra | 279 | 1,051 | 2,695 | 2,013 | 3 |  | 2 |  | 6 |  | 2 | 12 | 5 | 18 | 13 | 13 |
| Eland | 563 | 76 | 283 | 17 |  |  |  |  |  |  |  | 43 |  | 104 | 92 | 79 |
| Sable | 236 | 81 | 167 | 111 |  |  |  | 30 | 12 | 64 | 67 | 85 | 227 | 690 | 739 | 768 |
| Hartebeest | 169 | 133 | 421 | 96 |  |  | 15 | 5 | 9 | 6 | 125 | 141 | 200 | 538 | 527 | 532 |
| Hippo | 1,536 | 2,972 | 2,972 | 3,483 |  | 7 | 50 | 44 | 9 | 63 | 179 | 226 | 227 | 436 | 440 | 546 |
| Bushbuck | 43 |  |  |  |  | 5 | 84 | 40 | 154 | 31 | 354 | 386 | 235 | 2,210 | 1,995 | 1635 |
| Bushpig | 69 |  |  |  |  |  | 25 | 10 | 15 |  | 78 | 55 | 44 | 166 | 108 | 178 |
| Reedbuck | 58 |  |  |  | 12 |  | 175 | 138 | 380 | 156 | 2,052 | 2,648 | 1,981 | 11,807 | 10,434 | 10,176 |
| Grey duiker | 16 |  |  |  |  |  | 3 | 2 | 2 | 4 | 19 | 30 | 8 | 33 | 32 | 23 |
| Impala | 53 |  |  |  |  | 11 | 32 | 16 | 54 | 54 | 253 | 323 | 547 | 2,628 | 4,515 | 5,980 |
| Kudu | 206 |  |  |  |  | 24 | 6 | 5 | 18 | 46 | 115 | 159 | 187 | 1,169 | 1,403 | 1,898 |
| Nyala | 88 |  |  |  |  | 16 | 36 | 26 | 49 | 13 | 76 | 270 | 45 | 883 | 1,254 | 1,831 |
| Oribi | 17 |  |  |  | 1 | 41 | 58 | 40 | 246 | 45 | 583 | 1,107 | 257 | 4,404 | 3,833 | 3,936 |
| Red duiker | 13 |  |  |  |  |  |  |  |  |  | 10 | 5 | 6 | 19 | 20 | 19 |
| Warthog | 82 |  |  |  |  | 39 | 153 | 248 | 771 | 240 | 1627 | 2268 | 2328 | 8938 | 5316 | 10633 |
