# Supplementary material for: War-induced collapse and asymmetric recovery of large-mammal populations in Gorongosa National Park, Mozambique
Source: PLoS One. 2019 Mar 13;14(3):e0212864. doi: 10.1371/journal.pone.0212864 (PMC6415879; doi:10.1371/journal.pone.0212864)
Supplement: S5 Table — The table shows five records from the dataset; the interpretation of each column heading is described below. The full dataset of 70,102 spatially referenced sighting records is available as a supplementary online file (comma-separated values format) in S1 File. Readers should contact the corresponding author for additional information. (DOCX) [file pone.0212864.s009.docx]

**S5 Table. Illustrative metadata for the individual sightings of wildlife in Gorongosa National Park from aerial surveys spanning the period 1969–2018.** The table shows five records from the dataset; the interpretation of each column heading is described below. The full dataset of 70,102 spatially referenced sighting records is available as a supplementary online file (comma-separated values format) in **S6 File**. Readers should contact the corresponding author for additional information.

| **Id** | **Count**  **Year** | **Month** | **Observation platform** | **Latitude** | **Longitude** | **Within count strip** | **Rift** | **Within Rift in 2014 count block** | **Species** | **Number** |
| --- | --- | --- | --- | --- | --- | --- | --- | --- | --- | --- |
| 3564 | 2002 | November | Helicopter | -18.98930 | 34.53780 | 1 | 1 | 1 | Waterbuck | 2 |
| 3565 | 2002 | November | Helicopter | -18.99060 | 34.54520 | 1 | 1 | 1 | Warthog | 4 |
| 3566 | 2002 | November | Helicopter | -18.98960 | 34.54640 | 1 | 1 | 1 | Warthog | 16 |
| 3567 | 2002 | November | Helicopter | -18.99040 | 34.54420 | 1 | 1 | 1 | Buffalo | 26 |
| 3568 | 2002 | November | Helicopter | -18.98860 | 34.55390 | 1 | 1 | 1 | Bushbuck | 1 |

**Id:** consecutive number of observation (1 – 70,102)

**Count year:** year in which the aerial survey was conducted (1969 – 2018)

**Month:** month during which the aerial survey was conducted

**Observation platform:** helicopter or fixed-wing aircraft

**Latitude and Longitude:** as recorded by GPS during the flight (or digitized from count maps for Tinley 1969, 1970 and 1972)

**Within count strip:** “0” or “1” with “0” indicating an observation outside of the count strip (“0” not used for density calculations)

**Rift:** “0” or “1” for observation record that falls respectively outside or inside of the Rift Valley

**Within Rift in 2014 count block:** “0” or “1” for observation record that falls respectively outside or inside of the Rift Valley within the limits of the 2014-2018 count block

**Species:** animal species observed

**Number:** number of animals of a particular species for that particular sighting.
